# A review on the phytochemical and pharmacological properties of *Hyptis suaveolens* (L.) Poit

**DOI:** 10.1186/s43094-021-00219-1

**Published:** 2021-03-12

**Authors:** Pratibha Mishra, Saima Sohrab, Sanjay Kumar Mishra

**Affiliations:** Department of Botany, Ewing Christian College, Prayagraj, Uttar Pradesh 211003 India

**Keywords:** Lamiaceae, Pharmacological, Triterpenoids, Suaveolic acid, Ursolic acid, Main protease

## Abstract

**Background:**

Plants are the repository of variable number of valuable secondary metabolites that bears pharmacognostic and pharmacological implications having potentiality to emerge as super drugs in future. In-vivo production of these metabolites is influenced by the biotic and abiotic stresses resulting in continuous accumulation of diverse phytochemicals and their derivatives that can be useful in designing and developing potential drugs for future. The aim of the present study is to review the existence of medicinally important secondary metabolites and possible pharmacological and pharmacognostic importance of under-explored weed plant species *Hyptis suaveolens* (L.) Poit., to explore the potentiality of the plant for developing and designing the drugs for future.

**Main body of the abstract:**

*Hyptis suaveolens* belonging to family Lamiaceae is the rich source of medicinally important phytochemicals like essential oils, tannins, saponins, phenols, flavonoids, terpenoids, alkaloids, and sterols. One or many of these compounds have antioxidative, anti-inflammatory, antispasmodic, anti-septic, anti-cancer, anti-ulcer, antimicrobial, antibacterial, antiviral, antifungal, anti-diabetic, anti-fertility, diaphoretics, anticutaneous, anticatarrhal, antirheumatic, anti-ulcer, gastroprotective, immunomodulatory, analgesic, and antiviral activity.

**Short conclusion:**

*Hyptis suaveolens* contains unique terpenoid metabolites like suaveolic acid, suaveolol, methyl suaveolate, beta-sitosterol, ursolic acid, and phenolic compound like rosamarinic acid, methyl rosamarinate that have potentiality to substitute the traditional drugs as therapeutic agent against the resistant and newly emerged bacterial and viral pathogens. Pentacyclic triterpenoid, ursolic acid have been reported to have effective antiviral response against the SARS-CoV2 responsible for the present COVID-19 pandemic and HIV virus for which no effective vaccines are available till date. Ursolic acid has the ability to modulate the activity of main protease (M^pro^) that is essential for processing of SARS-CoV2 replicase-transcriptase machinery needed for viral replication and particle assembly.

## Background

The history of pharmacognostic and pharmacological implications of plants is intimately connected with the history of human civilization. A considerable number of plant-based drugs used today in modern system of medicine were used as crude drug source to cure human ailments by the ancient people as evident in ancient literature. Nature has established the parallel store house of plants as herbal remedies to cure human ailments. The modern pharmacopoeia provides the authentic information regarding the plant-based drugs, drug yielding plants, and their pharmacological effects on human body. The pharmacognostic value of plants is due to the presence of immense number of chemically variable secondary metabolites in plants. These pharmacologically active metabolites are produced in different plant parts at different stages of life cycle [1]. They provide additional advantages of promoting effective pollination and dispersal of seeds and other propagules or to protect the plants from the biotic and abiotic stresses [2].

The ever-changing climatic conditions not only affect the life cycle pattern, distribution, phenological, and phytosociological behavior of the plants but it also imposes noticeable stress on genomic organization and consequent change in the phytochemical profile of individual plant species growing under diverse conditions of environmental stresses. The imposed environmental stress affects the gene expression pattern and results in accumulation of diverse categories of secondary metabolites and its derivatives, changing the chemical profile within the individuals of a species growing under diverse ecological niches. These diverse secondary metabolites produced by the plants have ability to produce specific pharmacological responses making particular plant species a potential source of new medicine. The Indian subcontinent exhibits diverse climatic conditions due to prevailing geographical diversity which in turn largely controlled by annual monsoon, appears to be experiencing increasingly severe and erratic precipitation and resultant inter-specific and intra-specific diversity in the plant populations growing under different ecological habitats. Therefore, there is a need to analyze the characteristic chemical profile of the medicinally important plants growing under diverse climatic conditions and to understand how variation in temperature, moisture, and edaphic factors might affect the quality and quantity of pharmacologically valuable secondary metabolites. Some important phytochemicals found in plants are phenolics, alkaloids, terpenoids, glycosides, tannins, flavonoids, saponins, steroids, carbohydrates derivatives, gums, essentials oils, fatty oils, resins, mucilages, etc., that bear diverse pharmacological and pharmacognostic implications [3].

*Hyptis suaveolens* (L.) Poit. is one of the underexplored valuable medicinal plant used to treat various ailments in traditional system of medicine. It is an obnoxious weed of tropics and subtropics. The leaves of the plant are the source of pharmacologically important secondary metabolites having antispasmodic, anti-colic, anti-rheumatic, and anti-fertility properties [4]. The pharmacological significance of *H*. *suaveolens* is due to its sedative, diuretic, antispasmodic, aromatic, anti-inflammatory, anti-catarrhal, anti-cutaneous, anti-pyretic, ant-rheumatic, anti-soporific, and anti-cancer properties [5]. The essential oils contained in the leaves have potential antimicrobial and antifungal properties [6–8]. The root extract contains anti-retroviral compound called urosolic acid, a triterpenoid that may target retroviral integrases and proteases blocking the replication of retroviruses such as HIV [9, 10]. Most of these bioactive compounds found in *H*. *suaveolens* are used as therapeutic agent or as the precursors of useful drugs [11]. The mature leaves of *H. suaveolens* contain alkaloids as the major secondary metabolite followed by tannins and saponins respectively [12].

Family Lamiaceae or Labiatae popularly known as Mint family contains 236 genera and 6900–7000 species of aromatic plants [13]. Members of this family are herbs (perennial or annual herbs), shrubs, and trees. Stems are herbaceous rarely woody, prostrate and branched. Leaves are simple and oppositely arranged, rarely whorled, petiolate or sessile, hairy with characteristic aroma due to presence aromatic essential oils.

## Main text

### Taxonomic status of *Hyptis suaveolens* (L.) Poit.

*Hyptis* is one of the largest genus with more than 300 species of herbs, shrubs, and small trees, which belongs to the sub-family Nepetoideae under family Lamiaceae [14]. *H*. *suaveolens* (L.) Poit. is an annual or perennial herb exhibiting characteristic genotypic polymorphism and plasticity in morphological and physiological attributes [15]. It can grow luxuriantly in diverse environmental conditions due to the presence of variety of chemical compounds that provide it adaptive and survival advantages [16].

### Morpho-taxonomical features of *Hyptis suaveolens* (L.) Poit.

**Fig. 1:**
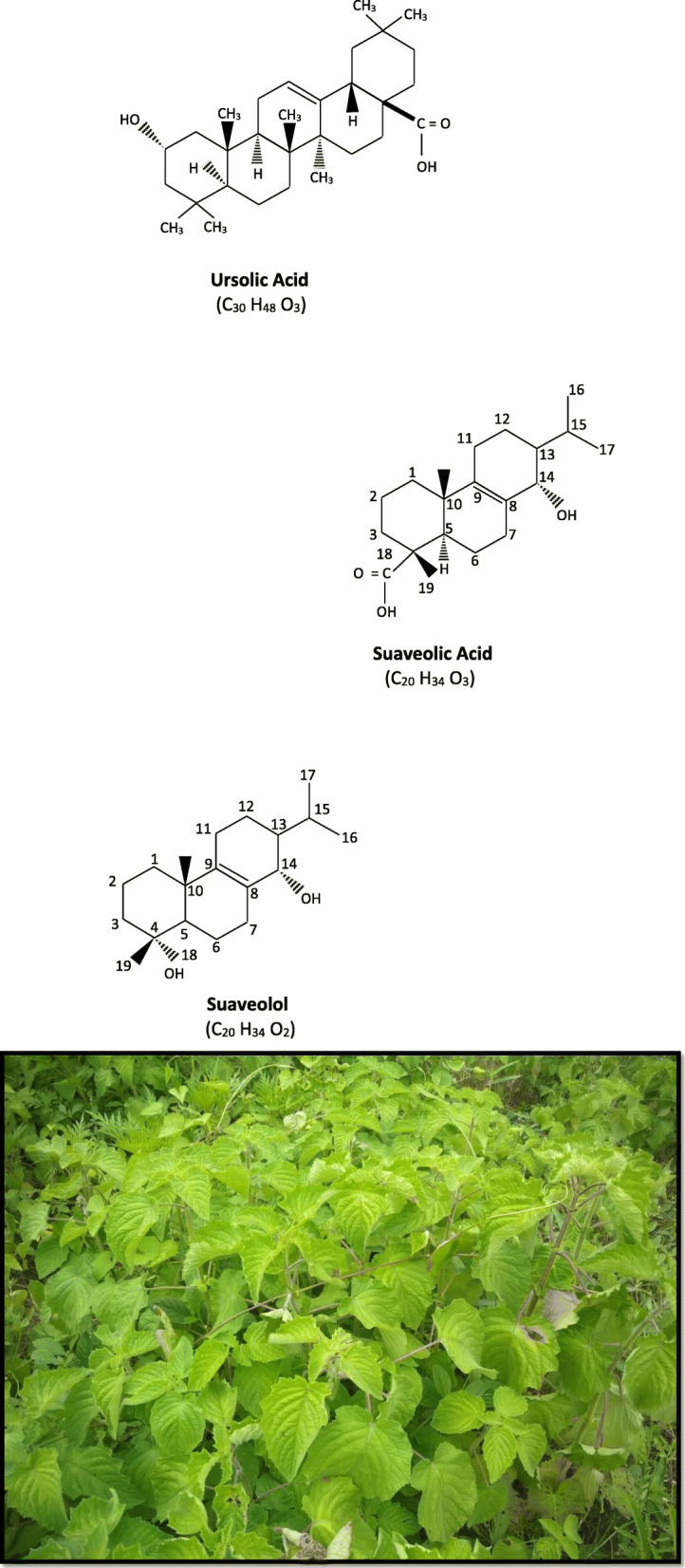
*Hyptis suaveolens* (L)

*H*. *suaveolens* (L.) Poit., popularly known as vilayati tulsi, bush mint, bush tea in India, is suffrutescent annual or perennial herbaceous weed growing along road sides. The stem is quadrangular, velvety, thick, and covered with long hairs and small erect glandular dots. Leaves are simple, opposite, decussate, and petiolate. The petioles are 2–4-cm-long bearing 2–10-cm-long and 4–6-cm-wide leaf blades having irregular serrated margin, covered with glandular hairs. Inflorescence is constituted of panicles or short umbels with 2–5 zygomorphic, blue purplish flowers arising in clusters at axillary positions. The campanulate, 5 teethed calyx ranges from 4 to 6 mm in length having glandular and pubescent surface marked by 10 ribs. The characteristic two-lipped corolla are blue in color exceeding the length of calyx tube, 8–10 mm long, lower lips are divided into 3 lobes, 4 didynamous stamens are inserted at the top of corolla tube, ovary divided into 4 lobes, and stylar end is filliform bearing small bilobed stigma at the top of the carpel [17] (Fig. 1).

### Phytochemical profile of *Hyptis suaveolens* (L.) Poit.

*H*. *suaveolens* is an aromatic medicinal herb characterized by the presence of diversity of phytochemicals like essential oils, phenolics, di and triterpenoids, steroids, flavonoids, etc. that constitute the chemical profile of the plants. *H*. *suaveolens* contain high amount of essential oils in oil glands, followed by alkaloids, flavonoids, tannins, phenolics, and saponins [11].

#### Phenolic compounds

Phenolics are the most abundant secondary metabolites synthesized by plants under biotic and abiotic stresses to adapt and survive it in the changing environmental conditions [18]. The phenolics are hydroxyl derivatives of ringed aromatic compounds produced by the plants under stress conditions and they may be categorized as phenolic acids if single ring is present and polyphenolics when more than one aromatic ring is present. The hydroxyl side chain present on the aromatic ring is responsible for the specific biochemical activity of phenolic compounds produced by the plants. Majority of plant phenolics occur as glycosides produced as a result of linkage of different phenolic rings with the sugar or acetylated sugar molecules [19, 20]. Depending upon the structure of aglycones, plant phenolics can be categorized as phenolic acid, flavonoids, polyphenolic amides, and other non-flavonoid polyphenols and their molecular mass ranges between 500 and 4000 Da [21]. The plant phenolics occur in both free and bounded form, the bounded phenolics constitute beta-glycosides through stomach and small intestine, and it reaches the colon in unaltered form where they exert their chemical bioactivity. The phenolic compounds are the potent antioxidants having the ability of scavenging free oxide radicals. The number and position of hydroxyl group and nature of substitution of aromatic rings confer the magnitude of antioxidative potential to the phenolics [18, 22]. Phenolics exhibits antioxidative property due to its redox potentiality, acting as reducing agent, free radical scavenger, hydrogen donors, metal chelator, quencher of lipid peroxidation, prevent DNA damage from oxidation, and scavenge reactive oxygen species, thus protecting the human body from free radicals [22].

Aromatic family Lamiaceae is characterized by the presence of diverse polyphenolic compounds like proanthocyanidins, prodelphinidins, profisetinidins, rosamarinic acid and methyl rosamarinate, caffeic acid, gentisic acid, p-hydroxybenzoic acid, vanillic acid, syringic acid, p-coumaric acid, protocatechuic acid, ferulic acid, chicoric, and caftaric acids in high concentration [23]. The leaves of *H*. *suaveolens* contain free polyphenols more abundantly than bound polyphenols, having higher antioxidant properties that are responsible for their protective effect from oxidative stresses by scavenging reactive oxygen species (ROS). Polyphenolics present in *H*. *suaveolens* have well established strong and effective antioxidative, hepatoprotective, cytoprotective, neuroprotective, and antimicrobial activity [24–27]. The Fe^++^ chelating ability of polyphenols protects human brain from iron induced lipid peroxidation [28].

#### Flavonoids

Flavonoids are the complex group of naturally occurring phenolics in plants, characterized by universal presence of phenyl-benzo-pyrone ring system. The flavonoids occur in free state or as glycosides (O-glycosides or C-glycosides) with its diverse derivatives like flavones, flavonol, flavanone, isoflavone, and chalcone anthocyanidins [29]. The flavonoids are present in glycosylated or esterified form, and are synthesized from derivatives of amino acid phenylalanine and acetic acid through shikimic acid pathway [29]. In plants, about 2000 types of flavonoids have been reported having antibacterial, antimicrobial, anti-cancer, anti-inflammatory, anti-diabetic, anti-aging, antiviral, cardioprotective activity, hence flavonoids are considered to be an important bioactive chemical constituent of pharmacologically reputed medicinal plants. It exhibits its anti-cancer activity by promoting apoptosis, induction of cell cycle arrest, proteasome inhibition, and by adversely interacting with carcinogenic enzymes [30]. Flavonoids acts as natural anti-inflammatory compounds by acting as potent inhibitor of transcription factors that modulate the expression of gene that produces inflammation inducer like cytokines, chemokines, and eicosanoids that results in inflammatory response by enhancing beta cell function of queretin-3-glucoside in diseases like, cardiovascular impairment, rheumatoid arthritis, leukemia, ileitis, sepsis, psoriasis, asthma, allergic rhinitis, and sclerosis [31, 32]. Flavonoids produces antioxidative responses by acting free radical scavenger and chelating metal ions such as iron and copper, inhibiting the activity of enzyme like microsomal monooxygenase, glutathione-*S*-transferase, mitochondrial succinoxidase, and NADH oxidase because these enzymes are involved in generation of free radical species (ROS); flavonoids are also helping in protecting lipids from oxidative damage caused by free radicals [33]. In addition to antioxidative potentiality by free radical scavenging, flavonoids exhibit antimicrobial activity. *Hyptis albida* contain flavanoids with hydroxy-methoxy flavones derivatives such as 5-hydroxy-7-4 dimethoxy flavones, 5-hydroxy-7-4-trimethoxy flavones, ermanin, nevadensin, and gardenin while in closely related *Hyptis suaveolens*, the flavonoid content ranges in between 10 and 13% such as gallic acid, Ferulic acid, quercetin, chlorogenic acid, and rutin [34, 35]. Many flavonoids such as apigenin, catechin, naringenin, quercetin, and rutin are known for their hepatoprotective activity and flavonoids such as hesperidin, apigenin, and aluteolin have anti-inflammatory and analgesic activity [36].

#### Terpenoids

Terpenoids are the oxygenated derivatives of hydrocarbon secondary metabolites produced by the plants that constitute isoprene units having characteristic pleasant aroma that forms volatile aromatic compounds called essential oils [37]. The essential oils are basically monoterpenoids or sesquiterpenoids and their oxygenated derivatives. The monoterpenes are basically derived from precursor geranyl pyrophosphate while sesquiterpenes are derived from farnesyl pyrophosphate and constitute one of the largest groups of secondary metabolites produced in plants under stress conditions [38].

Iwu et al. (1999) by GC/MS analysis reported 32 terpenoid compounds including 16 monoterpenes and 13 sesquiterpenes from the essential oil content of *H*. *suaveolens* having antifungal and broad spectral antibacterial activity against both Gram-positive and Gram-negative bacteria [39]. The diterpenoids comprise structurally diverse compounds that may be acyclic or having 1–5 ring system synthesized by mevalonate pathway and they constitute the integrated potential components of large number of medicinal plants of present and future drugs [38]. Suaveolic acid (14α-hydroxy-13β-abiet-8en-18oic acid) and suaveolol are two diterpenoid compound present abundantly in *H*. *suaveolens*, having high cytotoxic activity inhibiting growth of other plants adjacent to their clumps [40]*.* Plants specimens of *H*. *suaveolens* also contain three triterpene lactones and triterpenoid compounds like betulinic acid, ursolic acid, Beta sitosterol, oleanic acid, and acetyloleanic acid [34]. A new pharmacologically active pentacyclic triterpene Urs-12-en-3beta-29-oic acid have also been recorded from the *H. suaveolens* [41]. Diterpenoid endoperoxide, 13alpha-epi-dioxiabiet-8(14)-en-ol, present in petroleum ether extract of leaves of *H*. *suaveolens* have high antiplasmodic activity [42]. Ursolic acid (3-β hydroxy-urs-12-ene-28oic acid) is a natural pentacyclic triterpenoid (C_30_H_48_O_3_) which is an important terpenoids reported in *H*. *suaveolens* and other members of family Lamiaceae. It is synthesized from dammarenyl cation by cyclization of squalene in plants cells having diverse therapeutic potential and pharmacological effect [43]. The natural ursolic acid that occur in many plant species under family Lamiaceae need more analytical evidence to develop it into future drug to treat chronic diseases like cancer, diabetes, and viral infections like AIDS, COVID-19, etc. [43, 44].

By GS analysis, Poonkodi et al. (2017) reported that the presence of several medicinally important terpenoids in essential oil of *H*. *suaveolens* which include sabinene, trans caryophyllene, E-spathulenol, Rimuene, 1,8-Cineole, Beta-Elemene, Eucaliptol, Bergamotol, Z-alpha-trans, alpha-Selinene, Caryophyllene oxide, and alpha-humulene [45].

#### Alkaloids

Alkaloids are secondary metabolites, having therapeutic properties. They have heterocyclic ring system with basic nitrogen atom. About 5500 alkaloids have been reported. Alkaloids have analgesic, antiplasmodic, and antibacterial properties that is why they are used for medicinal purpose [46]. The leaves of *H*. *suaveolens* are rich in alkaloids having medicinal implications [11, 12].

#### Mineral elements

Different parts of plant contain calcium (Ca), potassium (K), phosphorus (P), nitrogen (N), magnesium (Mg), and Sodium (Na) in different concentrations while the ground tissue of leaves roots and stems also contains crystals of calcium oxalate [47].

### Pharmacological effects of *Hyptis suaveolens* (L.) Poit.

#### Antioxidative activity

Oxidative stress is a metabolic impairment that results in an abrupt imbalance between generation and detoxification of reactive oxygen species (ROS) in human body. The ROS constitute of variable reactive species (singlet oxygen, hydrogen peroxide, nitric oxide radicals, hypochlorite radical, super oxide anion radical, lipid peroxides, hydroxyl radical, etc.), acting as free radical that reacts actively with cellular proteins, enzymes, nucleic acids, and lipids results in cellular damage and invites different categories of chronic diseases and syndromes. Human body is provided with complex antioxidant protection system (APS) to mitigate the consequences of oxidative stress by neutralizing the free radicals [47].

*H*. *suaveolens*, a depository of diverse polyphenolics and flavonoid compounds having effective antioxidant property due to strong radical scavenging ability as determined by several analytical methods including ABTS (2,2′azino-bis-(3-ethylbenzothiazoline-6-sulfonic acid) and DPPH (2,2-diphenyl-1-picrylhydrazyl) [60]. Majority of plant species belonging to aromatic family Lamiaceae are rich source of polyphenolic compounds having antioxidative activity [48, 61, 62]. Some plant species under family Lamiaceae also contain antioxidant like vitamin C, vitamin E, quercetin, isorhammetin, kaempferol, etc. [63].

Flavonoids, a group of polyphenolic compounds, occur abundantly in plants and have potential antioxidative activity due to prevalence of hydroxyl groups and possess strong ability to scavenge free radicals produced due to antioxidative stresses [49, 64].

The most characteristic of plant phenolics and polyphenols is the capacity to scavenge ROS derived from lipids, proteins, oligo-nucleotides, and low-density lipoproteins (LDLs). These species are harmful for human health, cause oxidative stress-related chronic, and age-related diseases, such as cardiovascular disease (atherosclerosis), neuro-degeneration, (Alzheimer’s disease), carcinogenesis, and skin deterioration [65, 66]. Plant polyphenolic compounds acts as antioxidant by chelating metal ions like Fe(I), Fe(III), Cu(I), Cu(II). These ions are responsible for conversion of peroxides (H_2_O_2_) into aggressive hydroxyl anion by Haber Weiss reaction [67]. Poly-phenols block the action of superoxide generating enzymes like Xanthine oxidase and protein kinase C [68]. The poly-phenols exhibit their antioxidant action by hydrogen-atom transfer (HAT) and singlet-electron transfer (SET) mechanism. In the former, the phenolic functional group transfer its hydrogen atom to free radicals while in latter the transfer of single electron results in formation of radical cation [68].

The antioxidative potentiality of natural polyphenolics compounds with variable hydroxyl group is due to their ability to scavenge diverse ROS species by suppressing their formation by modulating the enzyme activity involved in their production [69]. Polyphenols like flavonoids may have the ability to react with non-polar compounds in the membrane lipid preventing the lipid oxidation and thus protect the membrane structure and function from oxidation [70].

#### Anti-cancerous activity

Cancer is a group of diseases that results in uncontrolled division of cell producing mass of abnormal cells. It is caused by change (mutation) in genetic material leading to uncontrolled cell division**.** Modern medicine provides number of treatment methods such as chemotherapy, radiation therapy, immunotherapy, hormonal suppression, and monoclonal antibody therapy, but these treatments have serious side effects. The essential oil of *H*. *suaveolens* containing terpenoids like sabinene, β-caryophyllene, trans-caryophyllene, Spatulenol, β-spathulenol, β-elemene, γ-elemene, Rimuene, α-humulene, Eucaliptol, 1-8-cineole, etc. as chief constituents show anti-cancer activity on MCF-7 cell line (cancer cell line of human breast) [45]. Ethanolic extract of *H*. *suaveolens* activates apoptosis process by inhibiting the activity of anti-apoptotic protein Bcl2 [71]. Ursolic acid can be employed as effective anti-cancer drug as it bears unique modulation effect on mitochondrial metabolism through multiple pathways promoting production of ROS that destabilize mitochondrial membrane potential, activating p53 pathway promoting apoptosis in cancerous cell [72]. The apoptosis as induced by ursolic acid involve caspase-dependent pathway of death of carcinoma cells [73]. Ursolic acid has the ability to target several signaling molecules involved in cellular transformation, cell proliferation, angiogenesis, and metastasis that results in cancer [50, 74–76]. Ursolic acid and related triterpenoids induces cell cycle arrest of cancerous cell lines by targeting carcinogenic enzyme through proteasome degradation [30]. Thus ursolic acid and its derivative can used as effective therapeutic agent against cancer.

#### Antibacterial activity

The flavonoids and phenolic compounds present in the essential oil of *H*. *suaveolens* exhibit strong antibacterial activity against pathogenic Gram-positive and Gram-negative bacteria such as *Staphylococcus aureus*, *Salmonella typhi*, *Pseudomonas aeruginosa*, *Lactobacillus plantarum*, *Escherichia coli*, *Vibrio vulnificus*, *Enterococcus fecalis*, and *Streptococcus fecalis* [26, 29]. But the antibacterial efficacy of phenolics and flavonoids present in essential oil of *H*. *suaveolens* is more pronounced in Gram-positive bacteria as compare to that of Gram-negative bacteria due to the presence of outer hydrophilic membrane in latter [53, 77, 78].

#### Antifungal activity

In human beings, there are number of diseases caused by fungi such as dermatitis, ringworm, athelete’s foot, aspergillosis, and mucormycosis [6, 7]. Essential oil from *H*. *suaveolens* shows strong antifungal activity against *Aspergillus* spp. (*A*. *flavus*, *A*. *parasiticus*, *A*. *niger*, *A*. *ochraceus*, *A*. *fumigatus*), *Saccharomyces cerevisiae*, *Mucor* sp., *Fusarium moniliforme*, etc*.* [54].

#### Anti-hyperglycemic activity/anti-diabetic activity

Hyperglycemia is the metabolic error in which glucose concentration increases in blood due to deficiency or insufficient production of pancreatic hormone insulin resulting in diabetes mellitus. Insulin deficiency in diabetic patient causes several abnormalities like accumulation of lipids (cholesterol and triglyceride). The methanolic extract obtained from the leaves of *H*. *suaveolens* exhibits anti-hyperglycemic activity in streptozotocin induced diabetic rats [55]. Ursolic acid a pentacyclic triterpenoid act as strong hypoglycemic agent enhances vesicular insulin transportation, secretion, and induces the uptake of insulin by the glucose transporter protein (GLUT4) located on plasma membrane by activating intracellular accumulation of calcium [79]. It also improves the insulin signaling in adipose tissue by enhancing the activity of beta cell function in streptoazotocin-induced diabetic mice [80].

#### Anti-fertility activity

Petroleum ether, alcohol, and aqueous extracts of *H*. *suaveolens* leaves show anti-fertility effect in pregnant rat. Studies showed that 100% anti-fertility activity shown by alcoholic extracts [81].

#### Antiplasmodial activity/anti-malarial activity

*H*. *suaveolens* is widely used in the treatment of malaria. It inhibits growth of both chloroquine-sensitive and chloroquine-resistant strains of *Plasmodium falciparum* under in vitro conditions. The chemical constituent responsible for this activity is dehydroabietinol that causes transformation of erythrocytes from discocytes to stomatocytes. Later, a diterpenoid 13alpha-epi-dioxiabiet-8(14)-en-18-ol found from petroleum ether extract of *H*. *suaveolens* leaves also shows antiplasmodial activity [82, 83].

#### Insect-repellent and Larvicidal activity

Mosquito-borne diseases such as dengue fever, yellow fever, malaria, filariasis, viral encephalitis affects large human population. Their bite causes serious allergy, local skin reaction, and systemic reaction. Extract of *H*. *suaveolens* shows larvicidal activity against yellow fever mosquito *Aedes aegypti* (L), *Aedes albopictus* larvae. Larvicidal activity of this plant is due to compounds like alpha-pinene, beta-pinene, sabinene, terpinolene, beta-caryophyllene, and 4-terpineol [84].

#### Wound healing activity

In wound healing process, a damaged tissue restored its normal state. It mainly depends on repairing ability of tissue. It occurs in three stages of inflammation, proliferation, and remodeling. In proliferative phase, angiogenesis (formation of new blood vessels from endothelial cells), collagen deposition, and wound contraction takes place. Alcohol, chloroform, ether, and petroleum extract of *H*. *suaveolens* showed wound healing activity by increasing hydroxyproline content, collagen deposition, dry weight of granulation tissue, and enhanced wound healing activity by increasing free radical scavenging action and by increasing the antioxidant enzymes in granuloma cells [85].

#### Anti-inflammatory activity

Anti-inflammatory activity shown by 2 diterpenes suaveolol, and methyl suaveolate against croton oil induced dermatitis of mouse ear. It induced dose-dependent edema inhibition [86]. The pentacyclic triterpenoid ursolic acid produces profound and effective anti-inflammatory effects [51]. The anti-inflammatory effect of *H*. *suaveolens* by scavenging free radicals similar to standard anti-inflammatory drug Ibuprofen was reported by several authors [52, 87].

#### Antiviral activity

Viral diseases are emerging as major cause of threat to human life resulting in severe death of human population worldwide. The frequent emergence of drug-resistant viral strains and acute shortage of effective vaccines and therapeutic agents for management and prevention of the viral infections invite the need of development of plant-based antiviral drugs. The plant-based natural pentacyclic triterpenoids are promising group of compounds synthesized by squalene cyclization that have notable antiviral property [88]. The aqueous alcohol extract of *H*. *suaveolens* containing pentacyclic triterpenoids shows better inhibition ability against Chickunguniya virus of Asian strain [89, 90]. The root extract contains antiviral compound ursolic acid that may act on HIV integrase, which prevent the replication of AIDS virus [9]. In Indian system of medicine, many plant members under family Lamiaceae are traditionally used for treatment of respiratory tract viral infection, cold, and fever [5]. Ursolic acid expresses its cytotoxicity and virucidal effect on rotaviruses by inhibiting the replication of viral particles and adversely effecting its particle maturation by targeting main viral proteins like VP6 and NSP2, that play crucial role in viral multiplication and pathogenicity thus by targeting early replication stages of viron, ursolic acid act as potent antiviral agent [91].

#### Miscellaneous pharmacological effects

The aqueous extract of the *Hyptis suaveolens* showed anti-ulcer activity against cysteamine hydrochloride-induced gastric and duodental ulceration by increasing healing of duodental ulceration and prevents the duodental ulceration in rats [92]. Hexane extract showed gastroprotective activity. The extract of aerial part of the plant *Hyptis suaveolens* prevented pyrogallol induced suppression of (humoral immune response (HIR) and cell-mediated immune response (CMIR) and also prevented the lipid peroxidation (LPO). The ethanolic extract of *H*. *suaveolens* showed toxicity effect on larvae of yellow fever mosquito *Aedes egypti.* Hydro-distillate leaves of *H*. *suaveolens* have showed acaricidal potency in ruminants [93]*.*

### *Hyptis suaveolens* as a source of potential therapeutic agent in COVID-19 treatment

The present COVID-19 pandemic engulfing millions of human lives crossing the territorial barrier is caused by SARS-CoV2, a spherical or pleomorphic enveloped nucleocapsid particle of β-coronavirus category bearing crown of human ACE2 receptor-specific glycoproteinaceous projections called spike. Its genome consists of a large single-stranded positive sense RNA associated with nucleoprotein. It infects the upper respiratory tract of human beings causing acute respiratory distress syndrome and resultant pulmonary failure and fatality in human beings [94–96].

The glycoproteinaceous spike protein of SARS-CoV play crucial role in viral pathogenesis. It is made up of two subunits S1 head and S2 filament protruding as club-shaped projections from the viral envelope bearing specific superficial binding affinity with ACE2 receptors present on the surface of human respiratory tract [56, 57].

When the viral particles enter the host cell, the viral envelope gets dissolved due to the acidic pH of autophagosome and viral genomic positive sense RNA enters inside the host cell. Replication and transcription of viral genome occur at cytoplasmic membrane involving coordinated synthesis of continuous and discontinuous RNA. The 5′ region of the SARS-CoV2 RNA that constitute the first open reading frame ORF 1a/b synthesizes a large polypepeptide, polypeptide a/b [58, 59]. These polypeptides are then processed by several virus-encoded proteases viz., chymotrypsin like-3CL^pro^, main protease-M^pro^-NSp5 and papain like NSP3 proteases into 16 nonstructural proteins (nsps) that constitute the viral replicase-transcriptase system consisting of helicases, RNA-dependent RNA polymerase, and other nonstructural proteins that are packed in membrane vesicles and involved in viral multiplication and assembly [97]. The natural ursolic acid (3β-3hydroxyl-urs-12-ene-28-oic acid, a pentacyclic triterpenoid) and its derivatives present in *Hyptis suaveolens* and other plants of family Lamiaceae act as potent antiviral agent against rotaviruses, HIV, influenza, and hepatitis B and C viruses [10, 53, 88, 98, 99].

Ursolic acid and pentacyclic triterpenoid act as strong protease inhibitor. The molecular docking simulation study based on integrated molecular modulation approach reveal that it act as potential inhibitor of main protease (M^pro^) of SARS-CoV2 [100, 101] and chymotrypsin like 3CL^pro^ protein [102, 103] that are produced after the processing polypeptide a/b and constitute the transcription replication machinery of SARS-CoV2. Thus the ursolic acid present in *H*. *suaveolens* can be used as therapeutic agent in targeting COVID-19 virus. The inhibitions of transcriptase-replicase enzyme adversely affecting the replication and assembly of corona viral particles and thus, ursolic acid and its derivative can emerge as potent therapeutic drug against COVID-19 virus and other life-threatening RNA viruses [104]. β-sitosterol also exhibit antiviral property against COVID-19 virus by targeting the receptor binding domain (RBD) of spike protein of virus interfering with the binding of viral spike protein with angiotensin-converting enzyme-2 (ACE2) receptor on human host cell, blocking the invasion of SARS-CoV2 virus inside the host cell [105].

## Conclusion

The present review tries to give broad and widespread assessment of medicinal value of underexplored, wild, and aromatic plant *Hyptis suaveolens* and future insight on its pharmacological expansion as effective therapeutic agent against metabolic and infective human disorders. The plant has well established antioxidative, anti-cancerous, anti-diabetic, anti-inflammatory, wound healing, cytotoxic, antiplasmodial, and antifungal properties due to the presence of diverse category of phytochemically active secondary metabolites having additive or synergistic pharmacological effects (Table 1). The presence of unique secondary metabolites like suaveolic acid, suaveolol, methyl suaveolate, β-sitosterol, ursolic acid, and phenolic compound like rosamarinic acid, methyl rosamarinate having potentiality to substitute the traditional drugs as therapeutic agent against the resistant and newly emerged bacterial and viral pathogens. Ursolic acid, pentacyclic triterpenoid compounds promote the apoptosis of cancerous cells by modulating diverse signaling molecules that induces cancer. It acts as protease inhibitor of main protease (M^pro^) and chymotrypsin like proteases (3CL^pro^) of SARS-CoV2 adversely affect its replication and particle assembly inside the host cells. Urosolic acid also has the ability to modify the integrase, a crucial enzyme needed for the establishment of HIV virus in human host cell. Thus secondary metabolites of *H*. *suaveolens* can be effectively exploited for the management of SARS-CoV2 responsible for present COVID-19 pandemic and HIV virus for which there is no effective vaccines have been developed till date. *H*. *suaveolens*, a widely grown weed plant, can be further explored and analyzed to develop potential, cost-effective drugs against ever mutating and resistant RNA viruses to save the humanity from deadly viral pathogens in future.

**Table 1:** Bioactive phytochemicals in *Hyptis suaveolens* and their pharmacological effects

Phytochemical constituents	Bioactive chemical compound	Pharmacological effect	References
Phenolics	Rosamarinic acid and Methyl rosamarinate	Antioxidative and anti-cancer property ( by acting as free radical scavenger, hydrogen donar, metal chelators), protects human body from free radicals and oxidative stress	[18, 22–27, 48, 49]
Flavonoids	Apigenin, Naringenin, Querecetin, Rutin	Hepatoprotective activity	[24, 35, 36]
	Hesperidin, Apigenin, Aluteolin	Anti-inflammatory and Anti-analgesic activity	[38, 39]
Terpenoids	Limonene, Diterpenoids Suaveolol, Suaveolic acid, 5 alpha-Androst-9(LL)-En-12	Antifungal activity, Antibacterial (Inhibits growth of Gram +ve and Gram -ve bacteria).	[39, 50–52]
	Diterpenoids 13alpha-epi-dioxiabiet-8(14)-en-ol	Antiplasmodic activity	[42, 53, 54]
	Alpha-pinene, beta-pinene, terpinolene, sabinene, beta cryophillene	Larvicidal activity against *Aedes albopictus* larvae.	[55]
	Urosolic acid	Antiviral activity against AIDS virus and SARC-CoV2 Virus	[5, 9, 44, 56–59]

## Data Availability

Not applicable.
